# Research integrity in Instructions for Authors in Japanese medical journals using ICMJE Recommendations: A descriptive literature study

**DOI:** 10.1371/journal.pone.0305707

**Published:** 2024-07-16

**Authors:** Shiho Koizumi, Kazuki Ide, Carl Becker, Tomoe Uchida, Miho Ishizaki, Akane Hashimoto, Shota Suzuki, Makiko Sano, Mayumi Toyama, Yoshitaka Nishikawa, Hiroshi Okada, Yoshimitsu Takahashi, Takeo Nakayama

**Affiliations:** 1 Department of Pharmacoepidemiology, Kyoto University Graduate School of Medicine and Public Health, Kyoto, Japan; 2 Center for Infectious Disease Education and Research (CiDER) Osaka University, Osaka, Japan; 3 Research Center on Ethical, Legal and Social Issues, Osaka University, Osaka, Japan; 4 Department of Preventive Medicine and Public Health, Keio University School of Medicine, Tokyo, Japan; 5 Department of Health Informatics, Kyoto University Graduate School of Medicine / School of Public Health, Kyoto, Japan; 6 Institute for Clinical and Translational Science, Nara Medical University Hospital, Nara, Japan; 7 Department of Social and Community Pharmacy, School of Pharmaceutical Sciences, Wakayama Medical University, Wakayama, Japan; 8 Department of Critical Care Nursing, School of Health Sciences, Bukkyo University, Kyoto, Japan; Institute of Medical Biochemistry Leopoldo de Meis (IBqM) - Federal University of Rio de Janeiro (UFRJ), BRAZIL

## Abstract

**Background:**

The International Committee of Medical Journal Editors (ICMJE) has published Recommendations for the Conduct, Reporting, Editing, and Publication of Scholarly Work in Medical Journals. These provide a global standard for writing and editing medical articles, including research integrity. However, no study has examined the research integrity-related content of Japanese medical journals’ Instructions for Authors. We therefore compared research integrity content in ICMJE member journals with those in the English- and Japanese-language journals of the Japanese Association of Medical Sciences (JAMS).

**Materials and methods:**

This was a descriptive literature study. We obtained Instructions for Authors from English- and Japanese-language journals listed on the JAMS website and the ICMJE member journals listed on the ICMJE website as of September 1, 2021. We compared the presence of 20 topics (19 in the ICMJE Recommendations plus compliance with ICMJE) in the Instructions for Authors, and analyzed the content of the conflict of interest disclosure.

**Results:**

We evaluated 12 ICMJE member journals, and 82 English-language and 99 Japanese-language subcommittee journals. The median number of topics covered was 10.5 for ICMJE member journals, 10 for English-language journals, and three for Japanese-language journals. Compliance with ICMJE was mentioned by 10 (83%) ICMJE member journals, 75 (91%) English-language journals, and 29 (29%) Japanese-language journals. The ICMJE Conflicts of Interest Disclosure Form was requested by seven (64%) ICMJE member journals, 15 (18%) English-language journals, and one (1%) Japanese-language journal.

**Conclusions:**

Although the topics in the JAMS English-language journals resembled those in the ICMJE member journals, the median value of ICMJE-related topic inclusion was approximately one-third lower in JAMS Japanese-language journals than in ICMJE member journals. It is hoped that Japanese-language journals whose conflict of interest disclosure policies differ from ICMJE standards will adopt international standards to deter misconduct and ensure publication quality.

## Introduction

Professional journals’ Instructions for Authors indicate the requirements and recommendations for writing articles. They generally include information on research integrity [1, 2]. The U.S. Office of Research Integrity defines research integrity as “active adherence to the ethical principles and professional standards essential for the responsible practice of research” [3]. When researchers write and submit papers, they need to clearly recognize research integrity and state their compliance with its rules, including ethical principles such as authorship and conflicts of interest (COI), and professional standards such as reporting guidelines and statistical statements [4]. Instructions for Authors can also reduce the number of poor-quality articles by raising contributors’ awareness of research integrity [5].

The International Committee of Medical Journal Editors (ICMJE) has published recommendations for authors who submit articles to member journals, including *Annals of Internal Medicine*, *The BMJ*, *Journal of the American Medical Association*, *The Lancet*, and *New England Journal of Medicine*. These recommendations have become the *de facto* international standard for many non-ICMJE member journals, which have voluntarily adopted them [6]. The latest version was revised in May 2023, and consists of four sections. Sections II–IV provide guidance on research integrity. Section II, “Roles and Responsibilities of Authors, Research Contributors, Reviewers, Editors, Publishers, and Owners,” focuses on publication ethics such as authorship and COI. Section III, “Publishing and Editorial Issues Related to Publication in Medical Journals”, focuses on research misconduct such as falsification, fabrication, and plagiarism. Section IV, “Manuscript Preparation and Submission” provides guidelines to improve the quality of articles, covering issues such as statistical presentation [6]. Each revision of the ICMJE Recommendations has added more information on research integrity [7].

In Japan, most Japanese Association of Medical Sciences (JAMS) societies have Japanese-language and English-language subcommittee journals. The editors of all the JAMS subcommittee journals established the Japanese Association of Medical Journal Editors (JAMJE) in 2008, developed Guidelines for Editing Medical Journals in 2015, and updated them in April 2022 [8]. JAMJE’s central issues were consideration of publication ethics and improving the quality of Japanese medical journals. The guidelines therefore reflect the situation in Japan and incorporate the ICMJE Recommendations [9].

However, little is known about the inclusion of research integrity topics in the Instructions for Authors of Japanese medical journals. These journals may not be consistent with international standards. This study therefore aimed to quantitatively compare the Instructions for Authors of ICMJE member journals with those of the JAMS subcommittee journals (both English- and Japanese-language), to clarify the content on research integrity.

## Materials and methods

### Study design

This was a descriptive literature study.

### Surveyed journals

We surveyed all ICMJE member journals listed on the ICMJE official website [10] (ICMJE member journals) and journals in the list of the JAMS subcommittee journals published in English (English-language journals) or Japanese (Japanese-language journals) [11] as of September 1, 2021.

### Eligibility criteria

We included all ICMJE member journals as of September 1, 2021 [10].

We included Japanese medical journals that: (1) were currently being published, (2) included Instructions for Authors on their websites, and (3) appeared on the official journal list of the JAMS subcommittee. The exclusion criteria for Japanese medical journals were: (1) out of print, (2) websites lacked Instructions for Authors, (3) not included among the JAMS subcommittee journals, or (4) being listed more than once because multiple societies of the JAMS publish the same journal.

### Evaluation of background information

The background information for the journals evaluated included: impact factor (IF, Clarivate) in 2020; domain (general, basic, clinical internal medicine, clinical surgery, and social); open access (OA); and publisher size. For IF, we searched for FY2020 IFs in Clarivate’s Journal Citation Reports [12]. Journals not included in the Clarivate database were categorized as not applicable. For domain, English-language and Japanese-language journals were categorized according to the list of the Japanese Medical Science Federation [13]. ICMJE member journal domains were classified using the description on each journal’s website. For OA journals, journals listed on the Directory of Open Access Journals website [14] by the Infrastructure Services for Open Access C.I.C., a non-profit organization in the United Kingdom, were counted as OA journals, following a previous study by Malički et al. [15]. Journals not included in the Directory of Open Access Journals database were categorized as not applicable. For publisher size, Taylor & Francis, Elsevier, Springer Nature, and Wiley-Blackwell were considered “major” publishers and all others “non-major” publishers, in line with a previous study [15].

### Topic selection

We identified topics to be surveyed from the ICMJE Recommendations. Sections II–IV contain research integrity topics, but no official checklist. To avoid arbitrary topic selection, we therefore first selected 15 topics described in the ICMJE Recommendations from the 19 topics used by Malički et al. [15]. Next, in consultation with a co-researcher (T. N.), we included four new topics from the ICMJE Recommendations: Authorship, Contributorship, Acceptable Secondary Publication, and Fees. We included Authorship because the ICMJE has recently emphasized its importance, adding new conditions for authorship in the 2013 revision [16]. Similarly, Contributorship is also an important research integrity topic that prevents ghost authorship and gift authorship [17]. We adopted Acceptable Secondary Publication to assess explicit exceptions to overlapping publications (not generally allowed) [18]. We inserted Fees because the ICMJE Recommendations require easy-to-find transparent statements of publication and submission costs [6], and because predatory journals often conceal or unexpectedly charge publication and submission fees [19, 20]. We also adopted three other topics, Compliance with ICMJE, Shared Authorship, and Use of Plagiarism Detection Software, not described in the ICMJE Recommendations but used by Malički et al. [15]. This gave 22 topics: 19 from the ICMJE Recommendations (15 from a previous study [15] and four unique to this study) and three topics not included in the ICMJE Recommendations but used by a previous study [15].

### Primary and secondary endpoints

Our primary endpoint was the median number of journals covering 20 topics: 19 from the ICMJE Recommendations, plus “Compliance with ICMJE,” used by Malički et al. [15]. We did not include the two topics of Shared Authorship and Use of Plagiarism Detection Software used by Malički et al. in our primary endpoint as these topics were not described in the ICMJE Recommendations.

Our secondary endpoints were (1) the proportion (%) of journals complying with the ICMJE Recommendations, and (2) the proportion (%) of instructions including COI descriptions, using content analysis [21]. The ICMJE Recommendations recommend submission of the ICMJE COI disclosure form [22] to all journals, not just those that are ICMJE members. We therefore investigated whether the journals mentioned: (1) use of the ICMJE Disclosure Form in their Instructions for Authors; (2) COI disclosure detailing exemptions other than the ICMJE Disclosure Form (e.g., precise monetary values or stock dividends); or (3) COI disclosure rules other than the ICMJE Disclosure Form (without describing exemptions).

### Topic definitions

We defined “description of research integrity” as the presence of topics in the Instructions for Authors. For each research integrity topic (19 topics included in the ICMJE Recommendations and three not included), we prepared topic-by-topic definitions (S1 Table). The topic evaluation was based on the presence or absence of descriptions that met the definitions for each topic. We did not include the number of words in the evaluation.

### Procedure

We evaluated each topic and COI content in three steps: 1) assessment by the primary evaluator (S.K.); 2) independent assessment by secondary evaluators (M.I., A.H., M.S., S.S., and T.U.); and 3) resolution by consensus for discrepancies in assessment. All evaluators were knowledgeable about research integrity, ICMJE Recommendations, and Instructions for Authors. Before the assessment, each evaluator showed a thorough understanding of the topic definitions and assessment processes.

### Statistical analyses

We determined the number of journals and the proportion (%) for each element of information, and the mean, standard deviation, median, minimum, and maximum values for IFs. For research integrity topics in the Instructions for Authors, we showed the mean, standard deviation, median, minimum, and maximum values for the total number of topics. For each topic, we indicated the number of journals and proportion (%) including that topic. As a secondary analysis, to compare the proportions of topics between the ICMJE member journals and Japanese- and English-language journals, we performed Fisher’s exact (two-tailed) probability test. For the content analysis of COI descriptions, we determined the number of journals and their proportions (%). We used JMP Pro ver. 16.1 statistical software (SAS Institute, Cary, NC, USA).

## Results

### Background information on the journals

**Fig 1:**
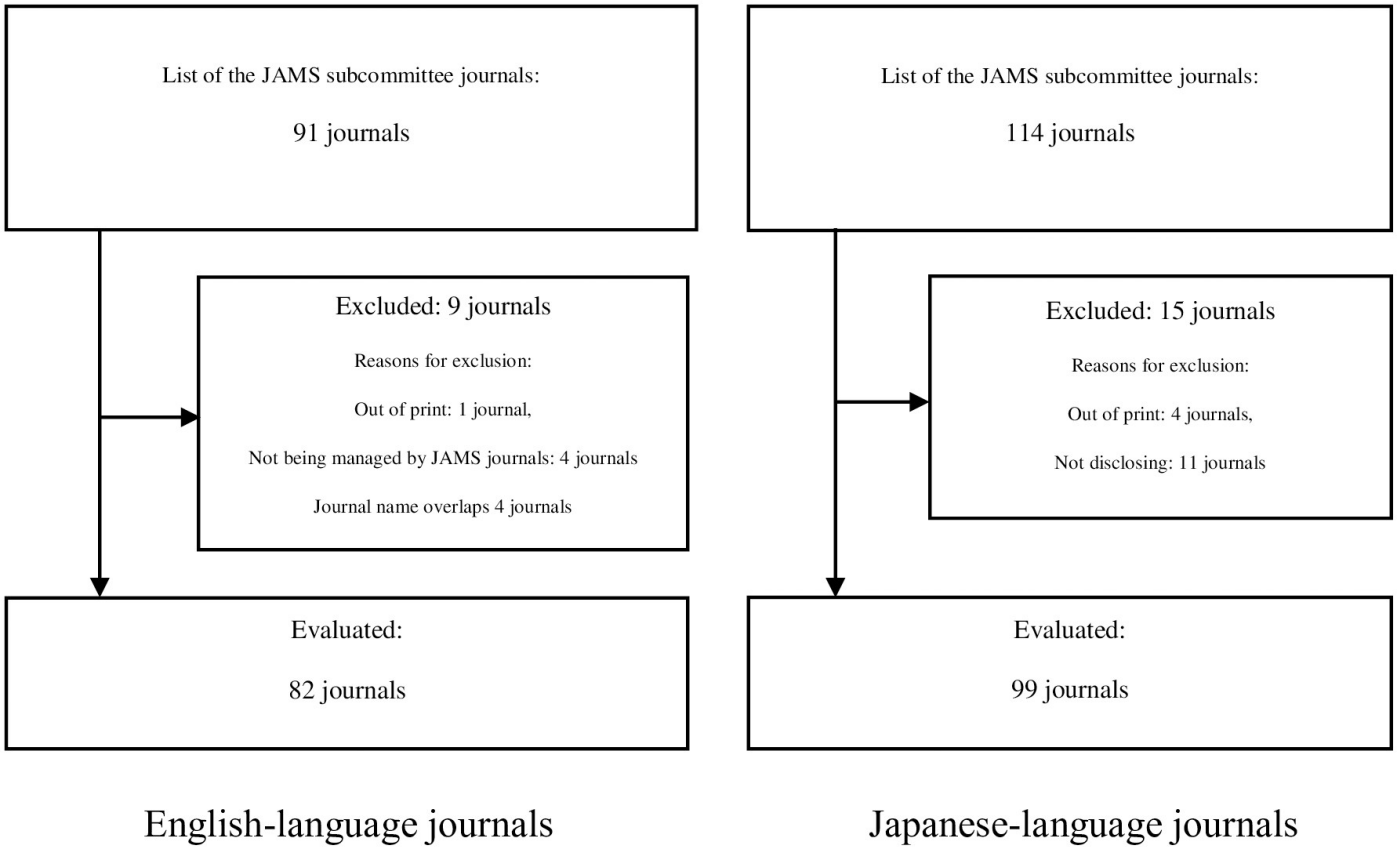
Flowchart of Japanese medical journal selections (English-language and Japanese-language journals).

Fig 1 shows a flowchart of the selection of English-language and Japanese-language medical journals in Japan. There were 91 English-language and 114 Japanese-language journals listed on the JAMS subcommittee journals website [11] on September 1, 2021, and we evaluated 82 English-language and 99 Japanese-language current non-overlapping journals. We compared these with the 12 member journals listed on the ICMJE website [10] on September 1, 2021.

Table 1 shows the background information for the evaluated medical journals.

**Table 1:** Background information for the evaluated medical journals.

		JAMS subcommittee journals	
	ICMJE member journals n = 12	English-language journals n = 82	Japanese-language journals n = 99
IF			
With IF	10 (83%)	66 (80%)	0 (0%)
Mean (SD)	30.8 (34.4)	3.5 (4.0)	NA
Median [Min, Max]	17.4 [0.6, 91.2]	2.7 [1.2, 33.0]	NA
Without IF	2 (17%)	16 (20%)	99 (100%)
Domain			
General	12 (100%)	0 (0%)	0 (0%)
Basic	0 (0%)	12 (15%)	9 (9%)
Internal medicine	0 (0%)	37 (45%)	43 (43%)
Clinical surgery	0 (0%)	27 (33%)	32 (32%)
Social	0 (0%)	6 (7%)	15 (15%)
OA journals			
Listed in DOAJ	5 (42%)	19 (23%)	2 (2%)
Publisher size			
Major	1 (8%)	58 (71%)	1 (1%)
Non-major	11 (92%)	24 (29%)	98 (99%)

Data are shown as n (%) unless otherwise indicated. Percentages may not sum to 100% because of rounding. DOAJ, Directory of Open Access Journals; IF, impact factor; OA, open access; SD, standard deviation.

### Research integrity topics, including those in the ICMJE Recommendations

Table 2 shows the results on all 22 topics on research integrity and each section of ICMJE Recommendations.

**Table 2:** Evaluation of research integrity topics, including those described in the ICMJE Recommendations.

	Research integrity topics	ICMJE member journals n = 12, (%)	JAMS subcommittee journals		P-value^a^ ELJ vs. ICMJE	P-value^a^ JLJ vs. ICMJE
			English-language journals n = 82, (%)	Japanese-language journals n = 99, (%)		
Total (22 topics)						
	Mean	10.5	10.8	3.9		
	Median [Min, Max]	11 [0, 19]	11 [4, 18]	3 [0, 14]		
19 topics described in the ICMJE Recommendations plus Compliance with ICMJE						
	Mean	10.1	10.0	3.9		
	Median [Min, Max]	10.5 [0, 17]	10 [4, 16]	3 [0, 14]		
Topics described in sections of ICMJE Recommendation						
Section II (6 topics)						
	Mean	3.6	3.9	2.0		
	Median [Min, Max]	4 [0, 6]	4 [2, 6]	2 [0, 5]		
1	Authorship	9 (75%)	51 (62%)	9 (9%)	0.53	<0.001
2	Contributorship	9 (75%)	40 (49%)	11 (11%)	0.124	<0.001
3	Peer Review Type	4 (33%)	60 (73%)	4 (4%)	0.016	<0.001
4	Null Results	2 (17%)	1 (1%)	0 (0%)	0.042	0.011
5	Conflicts of Interest	11 (92%)	82 (100%)	94 (95%)	0.128	0.51
6	Ethics Approval	8 (67%)	82 (100%)	82 (83%)	<0.001	0.24
Section III (8 topics)						
	Mean	3.7	4	1.4		
	Median [Min, Max]	4.5 [0, 6]	4 [0, 8]	1 [0, 6]		
7	Errata	4 (33%)	37 (45%)	2 (2%)	0.54	0.001
8	COPE	7 (58%)	54 (66%)	3 (3%)	0.75	<0.001
9	Image Manipulation	2 (17%)	31 (38%)	2 (2%)	0.20	0.057
10	Acceptable Secondary Publication	0 (0%)	9 (11%)	20 (20%)	0.60	0.119
11	Preprint	7 (58%)	37 (45%)	2 (2%)	0.54	<0.001
12	Fee	8 (67%)	64 (78%)	93 (94%)	0.47	0.012
13	Registration	9 (75%)	52 (63%)	17 (17%)	0.53	<0.001
14	Data Sharing	7(58%)	44 (54%)	2 (2%)	1.00	<0.001
Section IV (5 topics)						
	Mean	2.0	1.3	0.1		
	Median [Min, Max]	2 [0, 4]	1 [0, 4]	0 [0, 2]		
15	Reporting Guidelines	8 (67%)	38 (46%)	10 (10%)	0.23	<0.001
16	ORCID	2 (17%)	35 (43%)	0 (0%)	0.117	0.011
17	Limitations	5 (42%)	6 (7%)	1 (1%)	0.001	<0.001
18	Replication	4 (33%)	22 (27%)	1 (1%)	0.73	<0.001
19	Statistics	5 (42%)	4 (5%)	2 (2%)	0.001	<0.001
Topics not described in ICMJE Recommendations (3 topics)						
20	Compliance with ICMJE	10 (83%)	75 (91%)	29 (29%)	0.32	<0.001
21	Shared Authorship	3 (25%)	4 (5%)	2 (2%)	0.042	0.001
22	Use of plagiarism detecting software	2 (17%)	54 (66%)	0 (0%)	0.001	0.011

Data are shown as n (%) unless otherwise indicated. COPE, Committee on Publication Ethics; ELJ, English-language journals; JLJ, Japanese-language journals.

^a^ Fisher’s exact probability test (two-tailed).

The median number of ethical topics (19 topics from the ICMJE Recommendations plus compliance with ICMJE) included in Instructions to Authors was 10.5 in ICMJE member journals, 10 in English-language journals, and three in Japanese-language journals. For the secondary endpoint of compliance with ICMJE, 10 (83%) ICMJE member journals, 75 (91%) English-language journals, and 29 (29%) Japanese-language journals required compliance with the ICMJE Recommendations. The median number of topics included from each section of the ICMJE Recommendations was four for both ICMJE member journals and English-language journals, and two for Japanese-language journals for Section II (six topics); 4.5 for ICMJE member journals, four for English-language journals, and one for Japanese-language journals for Section III (eight topics); and two for ICMJE member journals, one for English-language journals, and none for Japanese-language journals for Section IV (5 topics). By major topic, nine (75%) ICMJE member journals, 51 (62%) English-language journals, and nine (9%) Japanese-language journals included the four conditions of authorship required by the ICMJE Recommendations in the Instructions for Authors. The Instructions for Authors in no (0%) ICMJE member journals, nine (11%) English-language journals, and 20 (20%) Japanese-language journals included the six conditions of acceptable secondary publication required by the ICMJE Recommendations.

Table 3 shows the results of another secondary endpoint, the content included in COI descriptions.

**Table 3:** Content of COI descriptions.

		ICMJE member journals n = 11, (%)	English-language journals n = 82, (%)	Japanese-language journals n = 94, (%)	Total n = 187, (%)
1.	Requires submission of ICMJE Disclosure Form	7 (64)	15 (18)	1^a^ (1)	22 (12)
2.	Has COI disclosure rules other than ICMJE Disclosure Form submission (describing exemptions such as precise monetary values or stock dividends)	0 (0)	27 (33)	90 (96)	117 (63)
3.	Has COI disclosure rules other than ICMJE Disclosure Form submission (without describing exemptions)	4 (36)	40 (49)	3 (3)	49 (26)

Data are shown as n (%) unless otherwise indicated. Percentages may not sum to 100% because of rounding.

^a^ Describes exemptions such as precise amounts of money or stock dividends

We evaluated 11 ICMJE member journals, 82 English-language journals, and 94 Japanese-language journals mentioning COI. Seven (64%) ICMJE member journals, 15 (18%) English-language journals, and one (1%) Japanese-language journal used the ICMJE Disclosure Form in their Instructions for Authors. No (0%) ICMJE member journals, 27 (33%) of English-language journals, and 90 (96%) of Japanese-language journals detailed exemptions (e.g., precise monetary values or stock dividends) other than the ICMJE Disclosure Form.

## Discussion

For the primary endpoints of the number of journals covering 20 topics (19 topics from the ICMJE Recommendations plus compliance with ICMJE), the median value was slightly lower for the JAMS English-language journals and substantially lower for the Japanese-language journals than for the ICMJE member journals. This pattern was generally the same for topics across Sections II–IV in the ICMJE Recommendations. For the secondary endpoint of ICMJE compliance, approximately 80% of the ICMJE member journals and nearly 90% of the JAMS English-language journals had submission rules stating compliance with the ICMJE Recommendations, whereas less than 30% of the Japanese-language journals had such rules. Regarding the other secondary endpoint, COI description inclusion (as determined using content analysis), more JAMS subcommittee journals had statements of exemption (e.g., monetary amounts and stock dividends) than ICMJE member journals, including 27 (33%) JAMS English-language journals and 90 (96%) Japanese-language journals.

The results for the primary endpoint suggest that JAMS English-language journals, of which 70% are published by major publishers, did not differ significantly from ICMJE member journals. Malički et al.’s previous study [15] found a higher number of research integrity topics in the world’s largest publishers’ Instructions for Authors. Major publishers publish multiple journals, and therefore their Instructions for Authors share many common research integrity topics. In addition, these major publishers produce journals with IFs. Resnik et al. [23] found that journals boasting higher IFs tend to have policies on data sharing, an integrity topic, incorporate shared data within the peer review process, and cite reproducibility as a justification for sharing data, indicating that IFs have a role in ensuring high journal research integrity. ICMJE member journals also had higher IFs as well as more research integrity topics. Therefore, although Japanese-language journals currently lack assigned IFs for linguistic reasons, it may be useful to establish domestic evaluation criteria for these journals in line with global standards. Language bias is an important issue that should be considered in relation to the possible low integrity of Japanese-language journals. Hugues et al. [24] found that the methodological quality of treatment effect estimates published in non-English language publications were worse than in English-language publications. A possible explanation is that higher quality studies tend to be submitted to English-language journals and lower-quality studies tend to be submitted to domestic language journals. This may indicate that English-language journals place more emphasis on research integrity than non-English language journals.

For one of the secondary endpoints, Compliance with ICMJE, we found that approximately 80% of ICMJE member journals and nearly 90% of JAMS English-language journals—but fewer than 30% of Japanese-language journals—have Instructions for Authors requiring compliance with ICMJE Recommendations. A large cross-sectional study [25] on Japanese physicians’ integrity in clinical research found that many Japanese physicians had low awareness of research integrity, including authorship, and may engage in inappropriate research practices such as copy-pasting, gift authorship, or omission of Institutional Review Board approval. To address Japanese researchers’ lack of awareness of research integrity, more Japanese-language journals should change their Instructions for Authors to comply with the ICMJE Recommendations, in line with the JAMJE Guidelines for Editing Medical Journals.

Our other secondary endpoint was the content of COI descriptions. JAMS English- and Japanese-language journals described exemptions such as money amounts and stock dividends (27 [33%] English-language journals and 90 [96%] Japanese-language journals) that were not described in ICMJE journals (0 [0%] journals). One possible reason for the large number of both English- and Japanese-language journals exempting fiscal details and stock dividends is that the JAMS COI Management Guidelines 2020 recommend that the COI status of all authors belonging to companies or organizations related to the published article be disclosed according to a prescribed form including standardized exemptions [26]. Approximately 30% of the JAMS English-language journals and more than 95% of Japanese-language journals follow these guidelines. However, the international standard ICMJE Recommendations recommend listing all COIs without exception, even for non-member journals. This creates a discrepancy with the COI Management Guidelines of the JAMS.

In May 2021, the COI Committee of the JAMS decided to recommend using the ICMJE Disclosure Form beginning in FY2022 [27]. However, the revised JAMS COI Disclosure Format published in 2022 [28] still provides exemptions, including precise monetary values or stock dividends. It is possible that this reflects Japanese-specific perceptions and practices regarding COI. There is some evidence that there are cultural differences across European countries in research integrity perspectives and practices [29]. However, in the context of more recent research culture and practice during the COVID-19 pandemic, the number of major medical and health science journals worldwide that have adopted the ICMJE COI policies for open science has increased [30]. It is hoped that JAMS English-language and Japanese-language subcommittee journals will widely adopt ICMJE Disclosure Forms in the near future, and that JAMJE will revise its COI guidelines to match. The ICMJE revises its COI disclosure standards on an ongoing basis, but has not established a COI management system [31, 32], and we suggest that the standards for COI disclosure should be debated worldwide. Our study has identified that COI disclosure standards in Japanese academic journals differ from foreign journals, suggesting that we should promote further international discussion and consensus about compliance with international standards. Topic Authorship was described in only about 10% of Japanese-language journals and 60% of the JAMS English-language journals. A 2011 questionnaire survey [33] of Japanese natural science researchers showed that fewer than 30% of their articles fulfilled the three conditions of authorship even for the *first* author. Some medical journals may have not updated their Instructions for Authors to include the latest four conditions, leaving the authorship conditions covering only the previous three conditions.

Japanese-language journals, which generally described the fewest number of research integrity topics, showed the largest number and percentage of inclusion of content on Acceptable Secondary Publication (20 journals; 20%) compared with English-language and ICMJE journals. One reason may be that some papers submitted to the JAMS English-language journals are translated into Japanese for later submission to Japanese-language journals. However, there was a case [34] in which a paper published in *The Lancet* was retracted because it had previously been published in a Japanese-language journal. To avoid inadvertent duplicate publication, Instructions for Authors of both English-language and Japanese-language journals should specify the conditions of acceptable secondary publication.

Descriptions of Reporting Guidelines, ORCID, Limitations, Replication, and Statistics were less common in Japanese-language journals than ICMJE member journals. The JAMS English-language journals were also less likely than ICMJE member journals to include the topics of Limitations and Statistics. Schriger et al. [35] faulted Instructions for Authors for inadequate descriptions of statistics and study methodology, such as replication. An increase in content of these topics is therefore desirable to improve the quality of medical research.

### Limitations

This study had three main limitations. First, the databases used as information sources for IFs and OA did not contain Japanese-language journals. It was therefore not possible to compare these issues across the three types of journals. Second, there were only 12 ICMJE member journals, and their background information differed from the JAMS English- and Japanese-language journals. However, the ICMJE Recommendations were originally developed for journals that are members of the ICMJE Committee, so we used them to compare English-language and Japanese-language journals. Finally, we did not investigate the number of characteristics or words in any descriptions in Instructions to Authors. Our evaluation of the content, quantity, and quality of the descriptions for each topic therefore remains preliminary. However, we believe our evaluators’ independent assessments, grounded in their common understanding, ensure a certain level of quality.

## Conclusions

Although the JAMS English-language journals resembled ICMJE member journals, the median ICMJE-related topic inclusion was approximately one-third lower in the JAMS Japanese-language journals than in ICMJE member journals. It is hoped that those Japanese-language journals whose conflict of interest disclosure policies differ from ICMJE standards will adopt international standards to deter misconduct and ensure the quality of publications in the future.

## Supporting information

S1 Table Definitions of 22 research integrity topics. (DOCX)

## Abbreviations

COI: Conflicts of interest
COPE: Committee on Publication Ethics
DOAJ: Directory of Open Access Journals
ICMJE: International Committee of Medical Journal Editors
IF: Impact factor
JAMJE: Japanese Association of Medical Journal Editors
JAMS: Japan Association of Medical Sciences
OA: Open access
SD: Standard deviation
