# Supplementary material for: Research integrity in Instructions for Authors in Japanese medical journals using ICMJE Recommendations: A descriptive literature study
Source: PLoS One. 2024 Jul 16;19(7):e0305707. doi: 10.1371/journal.pone.0305707 (PMC11251596; doi:10.1371/journal.pone.0305707)
Supplement: S1 Table — (DOCX) [file pone.0305707.s001.docx]

**Supporting information**

**S1 Table. Definitions of 22 research integrity topics**

| No. | Research integrity topics | Definitions |
| --- | --- | --- |
| Topics described in Section II, ICMJE Recommendations (6 topics) | | |
| 1 | Authorship | The Instructions for Authors state the four definitions of authorship in the ICMJE Recommendations (revised December 2019: (1) “Substantial contributions to the conception or design of the work; or the acquisition, analysis, or interpretation of data for the work;” (2) “Drafting the work or revising it critically for important intellectual content;” (3) “Final approval of the version to be published;” and (4) “Agreement to be accountable for all aspects of the work in ensuring that questions related to the accuracy or integrity of any part of the work are appropriately investigated and resolved.”^a^ |
| 2 | Contributorship | The Instructions for Authors clearly mention the specific role of each author. |
| 3 | Peer Review Type | The Instructions for Authors mention peer review type (open, single, double, or triple blind). |
| 4 | Null Results | The Instructions for Authors mention negative results publication. |
| 5 | Conflicts of Interest | The Instructions for Authors mention conflicts of interest. |
| 6 | Ethics Approval | The Instructions for Authors mention the requirement for ethics approval (e.g., IRB) or compliance with the Declaration of Helsinki. |
| Topics described in Section III, ICMJE Recommendations (8 topics) | | |
| 7 | Errata | The Instructions for Authors mention errata. |
| 8 | COPE | The Instructions for Authors mention the Committee on Publication Ethics (COPE). |
| 9 | Image Manipulation | The Instructions for Authors mention image manipulation. |
| 10 | Acceptable Secondary Publication | The Instructions for Authors state the six definitions of acceptable secondary publication in the ICMJE Recommendations (revised December 2019): (1) “The authors have received approval from the editors of both journals;” (2) “The priority of the primary publication is respected by a publication interval negotiated by both editors with the authors;” (3) “The paper for secondary publication is intended for a different group of readers; an abbreviated version could be sufficient;” (4) “The secondary version faithfully reflects the authors, data, and interpretations of the primary version;” (5) “The secondary version informs readers, peers, and documenting agencies that the paper has been published in whole or in part elsewhere” and “the secondary version cites the primary reference;” (6) The title of the secondary publication should indicate that it is a secondary publication (complete or abridged republication or translation) of a primary publication.”^a^ |
| 11 | Preprint | The Instructions for Authors mention preprints being accepted (or not) prior to publication. |
| 12 | Fee | The Instructions for Authors mention submission and publication fees. |
| 13 | Registration | The Instructions for Authors mention the need for study or protocol registration. |
| 14 | Data Sharing | The Instructions for Authors mention data sharing recommendations or the data sharing statement of ICMJE Recommendations (revised December 2019). |
| Topics described in Section IV, ICMJE Recommendations (5 topics) | | |
| 15 | Reporting Guidelines | The Instructions for Authors require or recommend compliance with the reporting guidelines. |
| 16 | ORCID | The Instructions for Authors recommend or require the use of Open Researcher and Contributor ID (ORCID). |
| 17 | Limitations | The Instructions for Authors mention the study’s limitations. |
| 18 | Replication | The Instructions for Authors mention reproducible methods. |
| 19 | Statistics | The Instructions for Authors mention not only statistical tests but also the confidence interval or effect size. |
| Topics not described in ICMJE Recommendations (3 topics) | | |
| 20 | Compliance with ICMJE | The Instructions for Authors mention compliance with ICMJE Recommendations. |
| 21 | Shared Authorship | The Instructions for Authors permit or do not permit joint authorship of more than one first author. |
| 22 | Use of plagiarism detecting software | The Instructions for Authors state that the editorial board may use plagiarism detection software. |

^a^ Cited from ICMJE Recommendations (revised in 2019).
